# Bridging the gap: Identifying diverse stakeholder needs and barriers to accessing evidence and resources for children’s pain

**DOI:** 10.1080/24740527.2022.2045192

**Published:** 2022-05-17

**Authors:** Nicole E. MacKenzie, Christine T. Chambers, Jennifer A. Parker, Erin Aubrey, Isabel Jordan, Dawn P. Richards, Justina Marianayagam, Samina Ali, Fiona Campbell, G. Allen Finley, Emily Gruenwoldt, Bonnie Stevens, Jennifer Stinson, Kathryn A. Birnie

**Affiliations:** a Department of Psychology and Neuroscience, Dalhousie University, Halifax, Nova Scotia, Canada; b Centre for Pediatric Pain Research, IWK Health, Halifax, Nova Scotia, Canada; c Department of Pediatrics, Dalhousie University, Halifax, Nova Scotia, Canada; d Solutions for Kids in Pain, Halifax, Nova Scotia, Canada; e Patient and Family Partner, Squamish, British Columbia, Canada; f Patient and Family Partner, Toronto, Ontario, Canada; g Northern Ontario School of Medicine, Thunder Bay, Ontario, Canada; h Department of Pediatrics, Women and Children’s Health Research Institute, Faculty of Medicine & Dentistry, University of Alberta, Edmonton, Alberta, Canada; i Department of Anesthesia and Pain Medicine, Hospital for Sick Children, University of Toronto, Toronto, Ontario, Canada; j Department of Anesthesia, Dalhousie University, Halifax, Nova Scotia, Canada; k Children’s Healthcare Canada, Ottawa, Ontario, Canada; l The Hospital for Sick Children and Lawrence S. Bloomberg Faculty of Nursing, Toronto, Ontario, Canada; m Department of Anesthesiology, Perioperative and Pain Medicine, University of Calgary, Calgary, Alberta, Canada

**Keywords:** knowledge mobilization, pediatric pain, stakeholder engagement

## Abstract

**Background:**

Stakeholder engagement in knowledge mobilization (KMb) activities can bridge the knowledge to action gap within children’s pain but may be influenced by how well stakeholder needs and barriers to evidence-based resources are addressed. The needs of different Canadian stakeholder groups related to children’s pain have not been examined, limiting the degree to which KMb efforts can be tailored to each group.

**Aims:**

The study aim was to identify shared and unique needs, barriers, and accessibility of evidence for children’s pain across three stakeholder groups: knowledge users (i.e., health professionals, administrators, policymakers, educators), researchers (including trainees), and patients, caregivers, and family members.

**Methods:**

This study comprised an online needs assessment survey. Analyses included descriptive statistics, one-way analyses of variances, and chi-square tests to examine differences between stakeholder groups. Open-ended responses were analyzed using conventional content analysis.

**Results:**

A total of 711 stakeholders completed the survey. Educational materials were the most utilized evidence-based resources among all stakeholders. Researchers and patients/caregivers/family members found resources significantly less accessible than knowledge users (*P* = 0.008). Knowledge of evidence was the primary barrier across all stakeholder groups (69.2%, *n* = 492); however, each group reported a need for stakeholder-specific resources. Finally, stakeholders desired opportunities to engage in the KMb process through partnerships and an increased awareness of children’s pain.

**Conclusions:**

Though stakeholders experience common barriers to evidence-based resources for children’s pain, their needs to address these barriers are diverse. Evidence-based resources should be tailored for stakeholders’ contexts, with diverse audiences in mind.

## RÉSUMÉ

**Contexte**: L'engagement des parties prenantes dans les activités de mobilisation des connaissances peut combler les lacunes entre les connaissances et l'action en matiére de douleur chez les enfants, mais peut être influencé par la mesure dans laquelle les besoins des parties prenantes et les obstacles auxquelles elles font face sont abordés. Les besoins des différents groupes de parties prenantes en matiére de douleur chez les enfants n'ont pas été examinés, ce qui limite la mesure dans laquelle les efforts de mobilisation des connaissances peuvent être adaptés àchaque groupe.

**Objectifs**: L'objectif de l'étude était de recenser les besoins communs et particuliers, les obstacles et l'accessibilité des données probantes en matiére de douleur chez les enfants dans trois groupes de parties prenantes: les utilisateurs des connaissances (c.-à-d. les professionnels de la santé, les administrateurs, les décideurs, les enseignants), les chercheurs (y compris les stagiaires) et les patients, prestataires de soins et membres de la famille.

**Méthodes**: Cette étude comprenait une enquête en ligne sur l'évaluation des besoins. Les analyses comprenaient des statistiques descriptives, des analyses unidirectionnelles des variances et des tests du chi carré afin d’examiner les différences entre les groupes de parties prenantes. Les réponses ouvertes ont été analysées à l'aide d'une analyse de contenu classique.

**Résultats**: Au total, 711 intervenants ont répondu au sondage. Le matériel pédagogique était la ressource fondée sur des données probantes la plus utilisée par toutes les parties prenantes. Les chercheurs et patients/prestataires de soins/membres de la famille ont trouvé les ressources beaucoup moins accessibles que les utilisateurs des connaissances (P = 0,008). La connaissance des données probantes était le principal obstacle dans tous les groupes de parties prenantes (69,2 %, n = 492); cependant, chaque groupe a fait état d'un besoin de ressources spécifique à chaque partie prenante. Enfin, les parties prenantes souhaitaient avoir l’occasion de s'engager dans le processus de mobilisation des connaissances par le biais de partenariats et d’une prise de conscience accrue de la douleur chez les enfants.

**Conclusions**: Bien que les parties prenantes rencontrent des obstacles communs aux ressources fondées sur des données probantes en matiére de douleur chez les enfants, leurs besoins pour s'attaquer à ces obstacles sont divers. Les ressources fondées sur les données probantes devraient être adaptées aux contextes des parties prenantes, en gardant la diversità des publics à l'esprit.

## Introduction

Pain impacts the life of every child; however, it remains undermanaged in both acute and chronic care contexts.^1–3^ Undermanaged pain can lead to significant physiological and psychological effects on children’s development, such as increased sensitivity to pain and fear of painful medical procedures.^4,5^ The undermanagement of children’s pain persists despite an abundance of evidence for its effective prevention and management.^6,7^ To promote the uptake of evidence to improve children’s pain, it is critical to engage diverse participants, such as patients, along with their caregivers and family members, researchers, and knowledge users (i.e., health professionals, administrators, policymakers, educators). These stakeholders can contribute to the production and uptake of scientific evidence through engagement in knowledge mobilization (KMb). KMb is the process by which evidence is made available through specific activities and tools^8^ and can bridge the knowledge to action gap that exists within children’s pain.^9^

The reach and impact of KMb activities in Canadian health care settings is influenced by the inclusion of diverse stakeholders in knowledge production and KMb resource creation. The absence of engagement of non-researcher stakeholders in KMb efforts results in significant research waste, including the development of very costly yet unavailable and unused KMb tools, including eHealth tools.^10^ Rather, concerted efforts that promote connections between researchers and other stakeholders are required to facilitate effective KMb and resource uptake.^10,11^ By engaging stakeholders in knowledge co-production and dissemination processes, access to evidence on pain management is made more attainable via existing connections to patients, caregivers, and their family members.^9^

To promote the reach and impact of KMb initiatives in Canadian health care settings, it is critical to understand the relevant needs and barriers stakeholders face to promote effective engagement on KMb initiatives. Needs assessments identify these barriers and needs by eliciting perspectives directly from stakeholders.^12^ Needs assessments increase the suitability of implementation initiatives by eliciting stakeholder characteristics and developing an understanding of how best to engage them in implementation.^13,14^ Known barriers to accessing evidence and resources for children’s pain management include a lack of knowledge about pain management, a lack of importance placed on pain management, and insufficient time to locate and utilize resources.^15–18^ Needs regarding access to evidence include further education on pain management as well as the need for, and awareness of, evidence-based resources, for clinicians and patients, caregivers, and family members.^15,19,20^ These barriers and needs have been identified within samples including a range of stakeholder types, including knowledge users (i.e., health professionals, administrators, policymakers, educators), researchers (including research trainees), and patients, caregivers, and family members; however, differences in the specific barriers and needs among these stakeholder types are not known. This missing perspective impedes effective dissemination and implementation of evidence-based practices to prevent and manage children’s pain. Understanding both shared and unique needs and barriers to accessing evidence would facilitate a tailored approach to disseminate and implement evidence in stakeholders’ own contexts.

The objectives of this study were twofold. The first objective was to examine the needs of three diverse stakeholder groups (i.e., knowledge users; patients, caregivers, and family members; researchers) regarding the development and organization of resources, as well as to examine barriers and facilitators to accessing evidence-based information on children’s pain. The second objective was to explore differences within these specific needs, barriers, and facilitators among these three distinct groups.

## Materials and Methods

### Study Design and Procedure

This study utilized both closed- and open-ended responses, where both types of data were integrated to provide a richer and more comprehensive understanding of the overall results. This study followed a traditional approach to needs assessment, an assessment that determines objectives to be addressed when establishing a project or study to promote tailoring of programs or recommendations.^21,22^ Data for needs assessments can be collected formally or informally, typically via surveys and interviews.^21,22^ This article aims to describe the methods used to gather the data with the intention of informing Solutions for Kids in Pain’s (SKIP) activities. The study was an online needs assessment survey developed and distributed by SKIP, a Networks of Centers of Excellence of Canada–funded knowledge mobilization network in Canada.^23^ SKIP’s mission is to improve children’s pain management by mobilizing evidence-based solutions through coordination and collaboration, with a vision of healthier Canadians through better pain management. This needs assessment survey was initially launched as part of SKIP’s funding application for the purpose of informing the development of their goals and activities in alignment with stakeholder needs. Advertising for survey participation was broad in nature, and study ads appealed to “any individual with an interest in children’s pain,” including knowledge producers (e.g., researchers, research trainees), knowledge users (e.g., health professionals, administrators, policymakers, educators), and end beneficiaries (e.g., patients, parents, caregivers). The survey was developed in English and translated into French, representing both official languages of Canada. Respondents provided consent to participate online prior to beginning the survey. Though data collection via this needs assessment was initially exempt from research ethics board review, because data collection was for assessment and improvement purposes,^24^ the use of these data for research purposes was later submitted for review and approved by the IWK Health research ethics board (REB# 1027199). A widespread, snowball sampling approach to recruitment was taken, with recruitment conducted via social media platforms (e.g., Twitter, Facebook, Instagram, etc.), e-mail, and listservs, and was open to Canadian and international respondents. For the purposes of the needs assessment, the term “pain” was used in an all-encompassing manner. SKIP’s focus is primarily on improving pain in children’s health institutions, and as such, “pain” in the current context referred to that which is commonly seen in health care settings, including both chronic and acute pain. The survey was available online from August 2018 to October 2020, with 93.1% (*n* = 662; 4.9%, *n* = 31 French-speaking respondents) of responses received within the first 3 months of survey launch (i.e., during the development of the initial SKIP network funding application to the Networks of Centers of Excellence of Canada). The survey remained open via the SKIP website until October 2020, although no active recruitment efforts were made after 2018. All data collected until October 2020 were included in the study analyses.

### Measures

The online survey (see Supplemental Material) consisted of 11 closed-ended and two open-ended questions regarding specific stakeholder needs for accessing evidence-based resources for children’s pain management, current evidence-based resources used, perceived accessibility of evidence-based resources, frequency and types of barriers experienced when accessing or implementing evidence-based resources, stakeholder type, and geographic location. Closed-ended questions consisted of Likert-scale and multiple-choice responses. Three closed-ended questions (i.e., inquiring as to resources used, needs, and barriers) also included an “other” response option, allowing respondents to elaborate with a free-text response. Respondents were asked two open-ended questions. The first question gauged respondents’ interest in knowledge mobilization activities (in collaboration with SKIP) related to children’s pain. The second open-ended question asked respondents to share other comments or feedback regarding a KMb initiative for children’s pain. The survey was developed in partnership with two patient/caregiver/family member partners (I.J. and D.P.R.), who were involved in developing the survey and ensuring the clarity and relevance of questions. The survey was pretested prior to launch with approximately ten stakeholders across the groups.

### Data Analyses

Data collected in French were translated into English, and all responses were analyzed together. This was deemed appropriate for two reasons. First, given that the survey questions were adopted into French (i.e., questions were directly translated from English), the presentation of information in each question was standardized and considered equal in comprehension and interpretation in both languages.^25^ Second, the French language data accounted for a minority of total survey responses (*n* = 30 of the 711 respondents included in the final analyses; 4.2%), with insufficient numbers and statistical power to consider meaningfully on their own.

Quantitative analyses were conducted to address both study objectives. To address objective 1, descriptive statistics were conducted to characterize general stakeholder needs, barriers, and accessibility of evidence-based resources. To address objective 2, a series of one-way analyses of variance were conducted to examine differences between the three stakeholder groups (i.e., knowledge users, patients/caregivers/family members, and researchers) in frequencies with which barriers are encountered when accessing evidence-based resources, as well as ratings of accessibility of evidence-based resources. Also related to objective 2, a series of chi-square tests was conducted to examine differences between stakeholder groups in terms of types of barriers encountered when accessing evidence-based resources as well as stakeholder needs to ensure uptake of evidence-based resources.

The open-ended qualitative data were analyzed to address the first study objective using conventional content analysis, an approach typically utilized to explore a concept of which little is known.^26^ The analysis was conducted with two coders (N.E.M. and O.P.) and followed standard content analysis procedures.^27^ Due to variability in the number of responses to each item from each stakeholder group, free-text data from all stakeholder groups were analyzed altogether. To compensate for the fact that all stakeholder open-ended data were collapsed for qualitative analyses, efforts were made to include examples from respondents within each stakeholder group within the results where appropriate. Both coders reviewed the responses to each of the five free-text response items to become familiar with the responses provided and used line-by-line coding for the first 25% of responses, categorizing responses into codes of like concepts using a qualitative data management software (NVivo 12, QSR International).^28^ Following initial coding, the coders met to discuss and refine the codes as necessary to ensure consistency in approach. Once the codebook had been refined, the first author (N.E.M.) coded the remaining responses and grouped the categories under higher-ordered headings, based on relationships between individual categories. These groupings were then abstracted into overall main categories that formulated a primary description of each topic. This process was carried out, as described, for each set of free-text responses. To ensure rigor in this analytic process, coders met frequently to discuss the codes and abstraction of the categories, an audit trail detailing analytic decisions was maintained, and quotes that illustrated the interpretations made from the abstraction analytic process were included.

The integration of the quantitative and open-ended qualitative data occurred through comparing the results within each topic and determining how concepts complemented or expanded on each other, following the steps outlined by Creswell and Plano Clark.^29^ The results were integrated by first reporting the quantitative statistical analyses, followed by the related qualitative findings, to facilitate comparison. A comment regarding whether the qualitative results converged with those of the quantitative findings was included to facilitate this direct comparison. Further integration was then conducted (see Discussion section, where common themes among the data are discussed in greater detail).

## Results

### Participants

A total of 711 stakeholders completed the survey, with 31 stakeholders (4.4%) responding in French. Survey respondents primarily identified as knowledge users (i.e., health professionals, administrators, policymakers, and educators; 62.2%, *n* = 442), followed by patients/caregivers/family members (27.2%, *n* = 194) and researchers (10.5%, *n* = 75). Stakeholders were predominantly from Canada (76.9%, *n* = 547), with 16.5% of responses from international respondents (*n* = 117). See Table 1 for complete demographic information.

**Table 1.** Participant demographics (*N* = 711)

	*n* (%)
Stakeholder type	
Knowledge users	442 (62.2)
Patient/caregiver	194 (27.2)
Researcher	75 (10.5)
Geographical region	
Canada	547 (76.9)
Alberta	107 (19.6)
British Columbia	82 (15.0)
Manitoba	10 (1.8)
New Brunswick	19 (3.5)
Newfoundland/Labrador	4 (0.7)
Nova Scotia	111 (20.3)
Ontario	175 (32.0)
Prince Edward Island	3 (0.5)
Quebec	20 (3.7)
Saskatchewan	15 (2.7)
Canadian territories	1 (0.2)
Outside of Canada	117 (16.5)
North America (United States)	55 (47.0)
Europe	34 (29.1)
Oceania	16 (13.7)
South America	9 (7.7)
Asia	1 (0.9)
Africa	2 (1.7)
Did not respond	47 (6.6)

Geographical region statistics are calculated as percentages within Canada and Outside of Canada, respectively; “Knowledge users” refers to health professionals, administrators, policy makers, and educators; “Researchers” includes trainees.

### Types of Evidence-Based Resources Used

#### Types of Evidence-Based Resources Used across All Stakeholders

Educational materials were the most commonly used resource among all respondents (27.0%, *n* = 192; see Table 2). This was followed by websites (23.9%, *n* = 170) and pamphlets (20.0%, *n* = 142).

**Table 2.** Type of evidence-based resources used (*N* = 711)

	Knowledge users^a^		Patient/caregiver		Researchers^b^			
	*n* (%)	Adjusted residual(%)	*n* (%)	Adjusted residual(%)	*n* (%)	Adjusted residual(%)	Chi-square value^c^	Significance (two-sided)
Pamphlets	101 (22.85)	2.46	24 (12.37)	−3.11	17 (22.67)	0.62	9.65	0.008
Educational material	131 (29.64)	2.03	31 (15.98)	−4.06	30 (40.00)	2.68	19.94	<0.001
Point of care tools	100 (22.62)	3.84	21 (10.82)	−3.15	9 (12.00)	−1.49	14.78	0.001
Websites	105 (23.76)	−0.12	47 (24.23)	0.12	18 (24.00)	0.02	0.02	0.992
Social media content	35 (7.92)	−4	36 (18.56)	3.5	12 (16.00)	1.23	16.32	<0.001
Apps	55 (12.44)	0.67	19 (9.79)	−1.02	10 (13.33)	0.43	1.09	0.579
Videos	53 (11.99)	−0.97	23 (11.86)	−0.53	16 (21.33)	2.29	5.25	0.073
Webinars	26 (5.88)	−0.63	14 (7.22)	0.6	5 (6.67)	0.13	0.42	0.81
Arts-based tools	37 (8.37)	1.79	9 (4.64)	−1.53	4 (5.33)	−0.61	3.24	0.198
Educational workshops	17 (8.76)	−2.87	79 (17.87)	2.7	11 (14.67)	−0.1	8.76	0.013
Policies	92 (20.81)	5.2	9 (4.64)	−4.85	8 (10.67)	−1.19	28.58	<0.001
Evidence summaries	49 (11.09)	0.6	8 (4.12)	−3.42	18 (24.00)	4.01	23	<0.001

Geographical region statistics are calculated as percentages within Canada and Outside of Canada, respectively. Percentage reported is within each stakeholder group who endorsed using each tool; *df* = 2; adjusted residual values significant at an absolute value greater than 1.96/−1.96.

^a^Knowledge users refers to health professionals, administrators, policymakers, and educators.

^b^Researchers includes trainees.

^c^Chi-square results significant at *P* < 0.05.

#### Differences in Types of Evidence-Based Resources Used by Stakeholder Type

Relative to patients/caregivers/family members and researchers, knowledge users used pamphlets significantly more, as well as point-of-care tools (i.e., evidence-based reference resources with information on quality of evidence and practice recommendations) and policies (see Table 2 for all results). Patients/caregivers/family members reported using social media content, as well as educational workshops, significantly more than knowledge users or researchers. No stakeholder group differences were reported for use of websites. Researchers reported utilizing evidence summaries (i.e., documents summarizing research on a given topic) more commonly than knowledge users and patients/caregivers/family members.

Webinars were the least commonly used resource collectively (6.3%, *n* = 45), with no significant differences across stakeholder type. There were also no significant differences between stakeholders on use of apps, videos, or arts-based tools (i.e., artistic expression of knowledge, such as visual arts).

#### Other Types of Evidence-Based Resources Used

Respondents shared some of the evidence-based resources for children’s pain they had used in response to the open-ended question. Information included here aligned with those categories endorsed in the quantitative data and provided specific examples of the types of resources used. Six subcategories of resource types were generated from this analysis (see Supplemental Table 1), including (1) electronic resources intended for patients/caregivers/family members (e.g., apps, videos, etc.), (2) literature intended for patients/caregivers/family members (pamphlets, posters, infographics, etc.), (3) knowledge user resources (e.g., clinical practice guidelines), (4) clinical tools (e.g., pain assessment questionnaires), (5) knowledge mobilization initiatives,^30,31^ and (6) training opportunities (e.g., scientific cafes, training sessions).

### Accessibility of Evidence-Based Resources for Children’s Pain Management

#### Accessibility of Evidence-Based Resources across All Stakeholders

Across all stakeholders, the mean rating of accessibility of resources was 4.80 (SD = 1.96), indicating moderate accessibility of evidence-based resources (0 = *resources were not accessible*; 10 = *resources are completely accessible*).

#### Differences in Accessibility of Evidence-Based Resources by Stakeholder Type

Stakeholders significantly differed in their perceptions of the accessibility of evidence-based resources, *F*(2) = 5.53, *p* = 0.004. Specifically, researchers found resources to be significantly less accessible than knowledge users (*M* = −0.62, SE = 0.25, *P* = 0.012, 95% confidence interval [CI] [−1.11, −0.14]). Patients/caregivers/family members also found resources to be significantly less accessible than knowledge users (*M* = −0.45, SE = 0.17, *p* = 0.008, 95% CI [−0.79, −0.12]).

### Barriers to Accessing/Implementing Evidence-Based Resources for Children’s Pain

#### Frequency of Barriers across All Stakeholders

**Figure 1.**
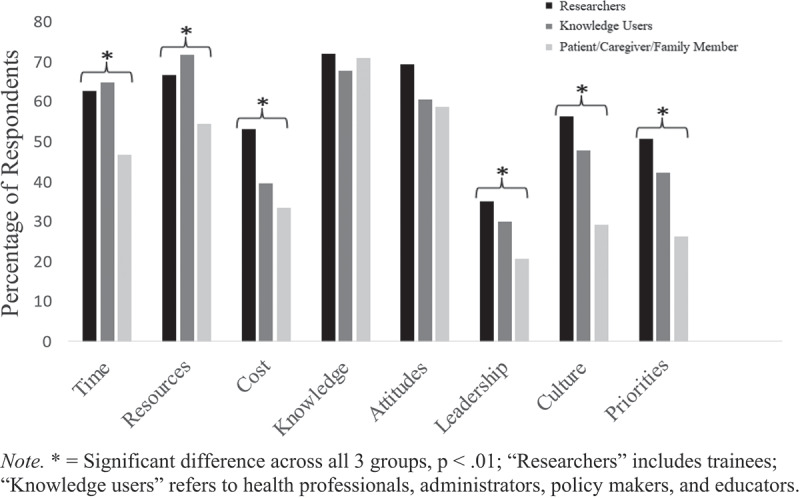
Barriers to evidence-based resources. Knowledge users refers to health professionals, administrators, policymakers, and educators. Researchers includes trainees. *Significant difference across all the groups, *p* < 0.01.

Respondents rated the frequency of barriers encountered on a scale from 0 to 10 (0 = *barriers are never experienced*; 10 = *barriers are experienced all the time*). Across all stakeholders, the mean frequency of barriers was 6.10 (SD = 1.93), indicating that stakeholders encounter barriers a moderate amount of the time when attempting to access or implement evidence-based resources. A lack of knowledge about children’s pain was the most common barrier reported among all stakeholders (69.2%, *n* = 492; see Figure 1).

#### Differences in Barriers Encountered by Stakeholder Type

There were no significant differences between knowledge users (*M* = 6.15, SD = 1.96), researchers (*M* = 6.10, SD = 1.86), or patients/caregivers/family members (*M* = 5.96, SD = 1.86) in reported barriers to accessing or implementing evidence-based resources, *F*(2) = 0.54, *p* = 0.583 (see Figure 1).

A lack of knowledge about children’s pain was the most common barrier encountered by patients/caregivers/family members (71.1%, *n* = 138) and researchers (72.0%, *n* = 54) and the second most common for knowledge users (67.9%, *n* = 300); however, stakeholders did not significantly differ on this barrier (see Table 3 for all results). Stakeholders also identified attitudes toward prioritizing children’s pain as a barrier to evidence-based resources, with no significant differences between stakeholders.

**Table 3.** Barriers to evidence-based resources (*N* = 711)

	Knowledge users^a^		Patient/caregiver		Researchers^b^			
	*n* (%)	Adjusted residual(%)	*n* (%)	Adjusted residual(%)	*n* (%)	Adjusted residual(%)	Chi-square value	Significance (two-sided)
Time	287 (64.93)	−3.69	90 (46.39)	4.41	47 (62.67)	−0.57	19.57	<.001
Resources	317 (71.72)	−3.76	106 (54.64)	4.11	50 (66.67)	−0.03	17.66	<.001
Cost	175 (39.59)	−0.15	65 (33.51)	1.96	40 (53.33)	−2.61	8.93	0.012
Knowledge	300 (67.87)	0.98	138 (71.13)	−0.68	54 (72.00)	−0.56	0.98	0.612
Attitudes	268 (60.63)	0.29	114 (58.76)	0.76	52 (69.33)	−1.56	2.62	0.269
Leadership	133 (30.09)	−1.71	40 (20.62)	2.63	25 (33.33)	−1.12	7.28	0.026
Culture	212 (47.96)	−3.01	57 (29.38)	4.68	41 (54.67)	−2.04	23.11	<.001
Priorities	187 (42.31)	−2.45	51 (26.29)	4.2	38 (50.67)	−2.23	19.52	<.001

Percentage reported is within each stakeholder group who endorsed using each tool; *df* = 2; adjusted residual values significant at an absolute value greater than 1.96/−1.96; chi-square results significant at p < .05.

^a^Knowledge users refers to health professionals, administrators, policymakers, and educators.

^b^Researchers includes trainees.

Knowledge users identified insufficient resources as the most common barrier to accessing or implementing evidence (71.7%, *n* = 317) and found this to be significantly more problematic than patients/caregivers/family members (see Figure 1). Knowledge users also identified time and culture as significant barriers, relative to patients/caregivers/family members and researchers. Researchers identified cost as a significant barrier relative to knowledge users and patients/caregivers/family members. Researchers identified a lack of leadership as a barrier to access or implementation of evidence significantly more than patients/caregivers/family members and knowledge users, despite it being the least commonly reported barrier among all stakeholders (27.8%, *n* = 198). Patients/caregivers/family members identified competing health-related priorities as a greater barrier to evidence-based resources relative to knowledge users and researchers.

#### Other Responses Regarding Barriers

In the “other” open-ended response category regarding barriers to accessing or implementing evidence-based resources, respondents described a range of barriers they had personally encountered with regard to both knowledge of resources and knowledge of the location of resources, as well as insufficient support to implement evidence. Two subcategories were generated from this analysis (see Supplemental Table 1), including (1) challenges locating and accessing relevant information and (2) lack of knowledge and support for accessing and implementing evidence-based resources.

In the first subcategory, *challenges locating and accessing relevant information*, respondents described a lack of knowledge of relevant resources and restricted access to resources, such as paid access publications. Respondents also described a lack of materials as a barrier for specific patient groups, health conditions, or non-English speakers, thus limiting the evidence available and its uptake. In the second subcategory, *lack of knowledge and support for accessing and implementing evidence-based resources*, respondents described a lack of both administrative and clinical leadership as a barrier to the implementation of evidence in clinical practice. This was further complicated by institutional policies related to implementation of evidence, such as paperwork and limitations on clinical practice due to lack of resources available to support implementation (e.g., limitations on optimal use of nurse-initiated pathways). Caregivers also described lacking knowledge of their rights to pain management, further complicating their ability to access and implement pain management resources. These access-related challenges converged with those reported in the quantitative data, in terms of the types of challenges confronted, and identify additional complications encountered.

### Needs to Ensure Use of Evidence-Based Resources

#### Analysis of Needs across All Stakeholders

Stakeholders identified a range of needs to ensure increased use of evidence-based resources. Tools for health professionals was the most frequently identified need among all respondents (52.0%, *n* = 370). Stakeholders identified a significant need for large-scale networks, such as SKIP, to aid in addressing these needs (*M* = 7.76; SD = 1.45; 0 = *not needed*; 10 = *very much needed*).

#### Differences in Needs by Stakeholder Type

A need for tools for health professionals was endorsed by knowledge users significantly more than patients/caregivers/family members and researchers (see Table 4 for all results). Knowledge users also reported a significantly greater need for educational opportunities compared to patients/caregivers/family members and researchers.

**Table 4.** Needs to ensure evidence-based resource use (*N* = 711)

	Knowledge users^a^		Patient/caregiver		Researchers^b^			
	*n* (%)	Adjusted residual (%)	*n* (%)	Adjusted residual(%)	*n* (%)	Adjusted residual(%)	Chi-square value	Significance (two-sided)
Identifying and collating current resources	154 (34.84)	1.61	50 (25.77)	−2.39	28 (37.33)	0.92	5.89	0.053
Centralizing access to resources	185 (41.86)	0.64	68 (35.05)	−1.95	38 (50.67)	1.81	5.87	0.053
Synthesizing knowledge in priority areas	121 (27.38)	1.85	25 (12.89)	−4.58	32 (42.67)	3.73	28.97	< .001
Engaging patients/caregivers	118 (26.70)	−5.87	94 (48.45)	4.65	36 (48.00)	2.52	34.45	< .001
Making existing tools more available	176 (39.82)	0.9	67 (34.54)	−1.34	31 (41.33)	0.53	1.87	0.393
Creating new tools	53 (11.99)	0.04	23 (11.86)	−0.05	9 (12.00)	0.01	0.003	0.999
Tools for patients	108 (24.43)	−2.71	82 (42.27)	5.2	9 (12.00)	−3.26	31.91	< .001
Tools for caregivers	127 (28.73)	−3.83	101 (52.06)	6.21	14 (18.67)	−2.97	41.5	< .001
Tools for health professionals	260 (58.82)	4.64	86 (44.33)	−2.52	24 (32.00)	−3.67	24.84	< .001
Tools for administrators and policymakers	59 (13.35)	2.1	16 (8.25)	−1.62	6 (8.00)	−0.98	4.43	0.109
French language tools	27 (6.11)	1.87	4 (2.06)	−2.16	4 (5.33)	0.17	4.75	0.093
Tools for underrepresented groups	119 (26.92)	−1.44	67 (34.54)	2.06	19 (25.33)	−0.71	4.31	0.116
Consultation for implementation	125 (28.28)	0.33	44 (22.68)	−1.86	29 (38.67)	2.21	6.99	0.03
Evaluation of implementation efforts	68 (15.38)	2.34	9 (4.64)	−4.09	16 (21.33)	2.24	18.72	< .001
Institutional support for ChildKind application	58 (13.12)	−1.54	16 (8.25)	−1.74	9 (12.00)	0.09	3.12	0.211
Educational opportunities	142 (32.13)	3.37	40 (20.62)	−2.59	15 (20.00)	−1.58	11.4	0.003
Media outreach for awareness	66 (14.93)	−3.31	46 (23.71)	2.1	21 (28.00)	2.18	11.6	0.003

Percentage reported is within each stakeholder group who endorsed using each tool; *df* = 2; adjusted residual values significant at an absolute value greater than 1.96/−1.96; chi-square results significant at p < .05.

^a^Knowledge users refers to health professionals, administrators, policymakers, and educators.

^b^Researchers includes trainees.

Researchers most frequently indicated a need for centralized access to resources (50.7%, *n* = 38); however, this need was not significantly greater than that of other stakeholders. Researchers also indicated a significantly greater need for synthesized knowledge in priority areas of children’s pain, as well as for consultation for implementation, relative to knowledge users and patients/caregivers/family members.

Patients/caregivers/family members also identified a need for tools for caregivers and a need for tools for patients significantly more than other stakeholders. They also indicated a need for engagement of patients/caregivers/family members significantly more than other stakeholders. Both patients/caregivers/family members and researchers identified a need for media outreach to raise awareness of evidence-based pain resources significantly more than knowledge users.

There were no significant differences between stakeholders on a need for identifying and collating current resources, making existing tools more accessible, creating new tools, tools for administrators and policymakers, French language tools, tools for underrepresented groups, or institutional support for Child Kind certification, which recognizes institutions who demonstrate excellence in pediatric pain care.^32,33^

#### Other Responses Regarding Needs

Respondents described practical needs to support implementation of evidence-based resources, including the importance of partnerships to create and support specific implementation plans. Three subcategories were generated to describe respondents’ feedback (see Supplemental Table 1), including (1) supportive strategies for effective evidence-based practice implementation; (2) broader awareness of, and support for, pain management practices and implementation; and (3) partnerships to promote development of knowledge mobilization initiatives.

In the first subcategory, *supportive strategies for effective evidence-based practice implementation*, respondents described that practical resources (e.g., staff, guidelines), as well as tailored supports for the given clinical environment stakeholders work in (e.g., availability of resources in different languages), were central to successful implementation. Training was also identified as key resource for health professionals to gain additional skills and for health trainees who are developing their practices around evidence-based pain management. In the second subcategory, *broader awareness of, and support for, pain management practices and implementation*, respondents described the necessity of a “culture shift,” where the value of evidence-based practice reflects in institutional policy. Increased public awareness of the importance of pain management, as well as knowledge of how to manage pain, was also cited as a key step toward promoting better implementation of evidence-based practices for children’s pain. In the third subcategory, *partnerships to promote development of knowledge mobilization initiatives*, respondents identified the importance of not only having diverse stakeholders contribute to developing resources for KMb but also having input from a range of stakeholders when creating implementation plans to add perspective relevant to each unique context where information may be utilized. These qualitative results converged with the quantitative data on specific stakeholder needs, further illustrating the need for strategies, support, and partnership opportunities to support implementation of the identified resources in each stakeholder group.

### Interest in Knowledge Mobilization Activities Related to Children’s Pain

Respondents described a range of interests in terms of contributing to resource development as well as disseminating evidence and resources. Three subcategories were generated to describe respondents’ feedback (see Supplemental Table 1), including (1) dissemination and implementation activities, (2) generating knowledge and resources, and (3) sharing perspectives as a stakeholder.

In the first subcategory, *dissemination and implementation activities*, respondents not only indicated interest in sharing resources within their own clinics and networks but also expressed interest in being the recipients of dissemination activities, such as trainings and gaining access to resources with consolidated evidence or information repositories. In the second subcategory, *generating knowledge and resources*, many respondents saw value in being able to partake in research activities, not only as investigators but as respondents as well. Other respondents expressed a desire to assist with creating resources, including those that could be made available in various languages. In the third subcategory, *sharing perspectives as a* *stakeholder*, respondents specifically discussed their interest in bringing their unique perspective as a stakeholder, as either a researcher, knowledge user, or patient/caregiver/family member, to engage in the knowledge co-production and/or evidence dissemination process. These qualitative results converged with the quantitative data presented by providing additional data on means by which stakeholders would like to be engaged to address the need for resources for specific stakeholder groups via KMb activities.

### Other Comments on Future KMb Needs and Directions in Children’s Pain

Respondents primarily described additional needs to support the management of children’s pain clinically, and they also identified pathways and partnership opportunities to support the goal of improved pain management through accessibility of evidence. Three subcategories were generated to describe respondents’ feedback (see Supplemental Table 1), including (1) children’s pain should be recognized and treated seriously, (2) the need for partnership to advance pain management efforts, and (3) development of further knowledge and resources for children’s pain.

In the first subcategory, *children’s pain should be recognized and treated seriously*, respondents stated the importance of having children’s pain management as a priority for clinical care and stated that SKIP as a network was a critical initiative to take meaningful steps to shift the focus onto uptake of evidence to promote pain management. In the second subcategory, *the need for partnership to advance pain management efforts*, partnerships between diverse stakeholders, as well as interdisciplinary partnerships, were discussed as crucial to not only creating evidence-based resources but supporting their implementation, when partners learn from each other to advance the goals of KMb in children’s pain. In the third subcategory, *development of further knowledge and resources for children’s pain*, respondents not only raised the importance of having further research evidence to support implementation and subsequent pain management but also highlighted the need for these resources to be developed with a range of families in mind. This included respondents who speak languages other than English, those from underrepresented populations (e.g., refugee families, Indigenous families), and children with special needs. These data converged with the quantitative results, further highlighting the identified barrier of pain management as lacking priority in clinical practice, as well as the identified need for more tailored resources for stakeholders.

## Discussion

In this survey of 711 stakeholders, the distinct needs of knowledge users (i.e., health professionals, administrators, policymakers, and educators), researchers (including research trainees), and patients, caregivers, and family members pertaining to evidence-based resources for children’s pain management were examined, as well as the barriers they face when trying to access these resources. Though stakeholders demonstrated similarities in the frequency and types of barriers faced, they differed significantly in their unmet needs to address these barriers and their preferred methods of accessing evidence. The qualitative results converged with the quantitative data, further highlighting specific needs and contexts in which barriers existed to accessing evidence-based resources for children’s pain management. These results ultimately inform how evidence may be best shared with stakeholders to improve the pain management of Canadian children.

### Stakeholder Preferences for Types of Evidence-Based Resource

Stakeholders significantly differed in terms of the types of evidence-based resources for children’s pain management they typically used. Overall, their preferences aligned with resource types already designed with their context in mind. For example, knowledge users used point-of-care tools significantly more than other stakeholders. Research shows that point-of-care tools are uniquely equipped to meet the information needs of knowledge users such as health professionals, given that such tools provide immediate access to evidence-based information for decision making.^34^ Researchers reported using evidence summaries significantly more than other stakeholders, which in a research context likely refers to published systematic reviews or meta-analyses. Researchers’ greater use of this particular type of evidence-based resource would facilitate their ability to remain up to date with a given field of literature, which is ever-changing.^35^ Finally, patients/caregivers/family members reported using social media and websites significantly more than other stakeholders. This finding is consistent with other research that shows patients and caregivers frequently use various social media platforms (e.g., Twitter, Facebook) to develop knowledge on medical conditions and general pediatric health.^36,37^ Knowledge of the modalities preferred by each stakeholder type can inform development of specific types of evidence-based tools designed to reach specific stakeholder audiences. It is clear that all stakeholders desire comprehensive yet concise, reliable data on which they can base their decisions for research and management of children’s pain management in Canadian health care settings. Finally, it should be acknowledged that the vast majority of these data was collected prior to the COVID-19 pandemic. As such, preferences for and reliance on online resources such as webinars may have changed and should be considered when developing educational resources.

### Stakeholders Differ in How Accessible They Find Evidence-Based Resources

In terms of access to evidence-based resources, stakeholders significantly differed in their perceived accessibility of resources, where researchers and patients/caregivers/family members reported resources as significantly less accessible than knowledge users. This finding is consistent with other research that shows that knowledge users, such as health professionals, can reliably access evidence via online means and demonstrate a greater awareness of evidence-based resources relative to other stakeholders.^38,39^ It is worth considering, however, that this finding may be influenced by the type of resources knowledge users have access to, where articles and resources behind paywalls are generally more accessible to health professionals relative to patients/caregivers/family members; that is, knowledge users may inherently have greater means to accessing resources, as well as a wider variety of resources available, relative to other stakeholder groups. Though researchers and trainees may also have greater access to publications behind paywalls, they reportedly lack familiarity with other evidence-based resources, such as clinical practice guidelines and other clinical resources, especially compared to knowledge users such as health professionals.^39^ It may be, however, that researchers seek evidence-based resources less frequently than other stakeholder groups, given that they do not typically implement evidence in clinical practice themselves and are less familiar with how to access them. This gives credence to the importance of KMb networks, such as SKIP, because they can offer support to close these competency and resource gaps. There is also evidence that when youth look online for information on their health and well-being, they encounter a great deal of misinformation or information that is not evidence based.^40^ This suggests that when patients/caregivers/family members are looking for their preferred evidence-based resources, identified earlier as social media or websites, they may struggle to identify or access high-quality information. Though each individual stakeholder group may differ in the degree to which they find resources accessible, the present findings shed light on a larger issue of accessibility of evidence for all stakeholders, where evidence may not be available where stakeholders traditionally seek information or the information available is not evidence based. Therefore, it is critical that evidence be made available in the locations where diverse stakeholders are known to seek it out, in an effort to engage in more meaningful dissemination.

### Stakeholder Barriers to Accessing Evidence-Based Resources

All stakeholders reported that, on average, they encountered barriers with moderate frequency. All stakeholders reported that a lack of knowledge about children’s pain was a significant barrier, as were attitudes toward prioritizing pain. This finding aligns with existing literature that has identified poor attitudes and knowledge, such as inaccurate beliefs that children forget pain, infrequent assessment of children’s pain, and not taking children’s reports of pain seriously, as being detrimental to the uptake of evidence for children’s pain management among knowledge users such as health professionals and caregivers/family members.^16,17,20,41^ Generating awareness and urgency about the importance of children’s pain and developing and disseminating resources serve as a foundation to dispelling misbeliefs and ultimately making evidence-based resources more accessible in Canada. Clearly communicating these key messages to one’s target audience is critical to effective KMb and drawing stakeholders’ attention to evidence-based resources.^42^

### Stakeholder Needs in order to Overcome Barriers

Though stakeholders largely agreed on the barriers encountered when accessing evidence-based resources on children’s pain, they significantly differed when it came to their needs. Knowledge users and patients/caregivers/family members identified a need for specific tools geared toward their respective stakeholder groups, whereas researchers identified a need for centralized access to resources and synthesized knowledge on children’s pain management. The finding that stakeholders have specific needs in terms of access to evidence-based resources aligns with other evidence that demonstrates that individuals require information specifically geared toward their individual needs.^43^ When information is delivered in a way that addresses stakeholders’ unique needs, the information can be more easily understood and implemented.^43^

In addition to these distinct needs in terms of resource types, stakeholders discussed the need to develop and gain support for implementing evidence-based practices. Specifically, stakeholders identified the need for more partnerships between diverse stakeholder groups for the development, dissemination, and implementation of KMb resources, where they could impart their knowledge and unique perspectives at various stages of the KMb process. The desire to be engaged with multiple stakeholders, including patient partners and interdisciplinary health professionals, is a critical component of the evidence uptake process, because research has identified a lack of stakeholder engagement to be a significant barrier to evidence uptake.^44^ Therefore, not only do stakeholders express a preference to engage in partnership in the knowledge production and dissemination process but evidence also suggests this may lead to more effective uptake of evidence in the Canadian health landscape.

### Recommendations for Future Action

#### Tailor Evidence-Based Resources

Though stakeholders identified common barriers to accessing evidence-based resources for children’s pain, in terms of both type and frequency, the needs that stakeholders reported to address these barriers differed significantly. It is recommended that resources be tailored for different stakeholder groups, not only in terms of evidence-based resources themselves but also in terms of how and where these resources are disseminated. Tailoring is a method of adapting processes and supports based on stakeholder needs, as determined through stakeholder engagement in evidence review and uptake.^45,46^ Tailoring efforts should consider the most appropriate strategy for KMb as informed by the identified needs and barriers, potential adjustments of strategies given the stakeholder’s context, and consideration of resources required to facilitate the KMb activity,^13^ especially in the Canadian health care context. Furthermore, tailoring not only involves adapting the type of content communicated to the intended stakeholder audience but also involves attending to the language used (e.g., plain versus technical language) and where the resource is made available.^47^ When KMb resources are tailored to individuals’ needs, they are more apt to be effectively disseminated and used.^48^

#### Create Evidence-Based Resources for Diverse Audiences

Based on the present findings, it is recommended that tailoring also extend to evidence-based resource development for diverse Canadian audiences. These include stakeholders who are non–English speaking as well as any adaptations that are needed to ensure equitable and inclusive to reach marginalized or racialized populations or population groups based on culture, ethnicity, or other factors. Based on the present findings, there is a need for evidence-based resources on pain management for diverse audiences that have been developed in different languages (e.g., French, Arabic), for underrepresented populations (e.g., refugee families, Indigenous families), and for children who are neurodevelopmentally diverse (e.g., autism spectrum disorder). Tailoring resources to meet these unique needs is especially important given that these groups may have specific needs or questions when it comes to knowledge around children’s pain management.

#### Foster Partnership between Stakeholders When Developing and Disseminating Evidence-Based Resources

The findings of the present study extended beyond simply identifying stakeholder needs by shedding light on stakeholders’ desired involvement in this process. In order to address this desire, it is recommended that stakeholders be engaged in partnership during the development of such resources. Partnership with stakeholders will support resource development and dissemination by shedding light on preferred modalities of information delivery within the given stakeholder group but can also promote greater uptake and influence of the resources that are subsequently developed.^11^ Stakeholders in the present study clearly identified a desire to be engaged in KMb activities through sharing perspectives in the knowledge production and resource creation stages, partnering with diverse stakeholders during this process, and participating in disseminating such resources within their own networks. Engaging stakeholders, including various types of knowledge users (e.g., health professionals, policymakers, etc.) and patients/caregivers/family members, at various stages of KMb activities is a key recommendation because it can facilitate the tailoring process by sharing specific needs, preferences, and critical perspectives on how information can be best presented as well as implemented in a Canadian context.^45,49^ In turn, partnership between stakeholders at various points in the KMb process can serve to support efforts to address the specific needs and barriers of stakeholders. To engage stakeholders in partnerships within KMb processes is to work in line with the integrated knowledge translation approach, where all relevant stakeholders are engaged in the knowledge co-creation process such that relevant needs are integrated within the broader knowledge production, dissemination, and implementation phases.^13^ Integrated knowledge translation therefore creates another opportunity to address the needs identified in this study, including that of partnership, where stakeholders would be embedded in KMb projects at various stages. Establishing these engagement and partnership opportunities can be facilitated through networks such as SKIP, which can form bridges between stakeholders who can partner together to ultimately create more meaningful and relevant KMb outcomes,^50^ such as evidence and resources.

#### Communicate Strategies and Provide Support for Implementation

Implementation activities take a step beyond dissemination and represent the stage at which evidence is ultimately adopted within a setting, such as a hospital or clinic.^47^ In the present study, stakeholders reported that beyond simply having access to resources specific to their needs and preferred modalities, it was important to have supports available for using the evidence for pain management in practice. Therefore, to support use of evidence in clinical settings, it is recommended that stakeholders be provided with practical recommendations for implementing evidence, as well as educational resources, training opportunities, and other practical resources, such as staff responsible for evidence uptake in clinical settings. When the intention of evidence is clearly communicated and justified, sufficient training and information are provided, and there is adequate team support in the clinical environment where the evidence will be used, evidence is more likely to be successfully integrated as a part of standard pain management for children.^51,52^ Therefore, this information should accompany evidence-based resources, such that stakeholders are fully supported in their efforts in both accessing and utilizing evidence. SKIP is committed to supporting implementation efforts, communicated via their vision of healthier Canadians through better pain management for children, and long-term outcomes of improved children's pain management in Canadian health institutions (see kidsinpain.ca). SKIP’s activities to accomplish these goals is driven by engagement in a range of evidence-based dissemination and implementation strategies, such as those summarized by Tutelman and colleagues^53^ and Chambers.^9^ This commitment is underpinned by the current study findings, reinforcing the importance of an organization such as SKIP to support the identified needs for implementation to improve outcomes within children’s pain management.

### Strengths and Limitations

This study is the first of its kind to examine barriers and needs for accessing evidence-based resources for children’s pain management and is strengthened by its large sample size and integration of closed- and open-ended data. This approach facilitated identification of the specific needs and barriers experienced by stakeholders and facilitated an understanding of how they believed these needs could be most effectively met in detail to provide recommendations to researchers in future KMb endeavors. This study was not without its limitations. Firstly, the sample distribution comprised predominantly knowledge users, which means the needs, barriers, and accessibility issues reported by knowledge users may have skewed the findings and overshadowed those expressed by patients/families/caregivers and researchers. Further to this, the knowledge user stakeholder group was defined as potentially including a variety of professionals, including health professionals, administrators, policymakers, and educators. As such, results may not be directly relatable to each type of professional within this group, and future research is needed to account for the unique needs of the stakeholders that exist within this broader category, including policymakers and other decision makers. Related, trainees were also captured within the researcher stakeholder group; however, there was potential heterogeneity among the trainees represented in the current sample, such as undergraduate, graduate, or postdoctoral trainees; fellows; and residents. Furthermore, career stage was not considered within those who identified as a career researcher and, as such, researchers at various career stages may have been represented within the sample. The needs of these diverse trainees and researchers may be distinct; therefore, future research should consider exploring the unique needs of the specific stakeholders within these groups so they are appropriately supported in KMb activities. In addition, the data collected via this survey did not explore the specific contexts in which stakeholders were attempting to access or utilize evidence-based resources for children’s pain. The context in which evidence is implemented can influence outcomes by way of factors including finances, support, and leadership.^53^ Therefore, conclusions around the role of contextual factors related to barriers or needs cannot be accounted for. Related, pain was not defined as part of the study introduction and, as such, respondents likely drew on diverse experiences with pain in their clinical and personal experiences (e.g., chronic pain clinic, acute pain services, etc.). As such, given the broad sample of respondents described in this study, there may be nuances in stakeholder needs, barriers, and facilitators within specific pain services that are not captured by the current study results. Furthermore, because of the nature of this needs assessment, minimal demographic data were collected from respondents, thus limiting the examination of these results in the context of various demographic variables (e.g., ethnicity, gender, socioeconomic status, etc.). Future research should explore demographic factors as well as the contexts in which stakeholders access and use evidence to enrich understanding of how they can be best supported in these unique contexts. This study did not explore perspectives from policymakers or other decision makers. Given the distinct roles of these stakeholders relative to those included in this study, the generalizability of these findings is limited in terms of how they relate to these other audiences. The present study is limited by factors related to data collection. Recruitment primarily occurred via social media, which may have introduced bias into the present findings, given that respondents may have been savvier with online connectedness and knowledge sharing, which could relate to the ease with which they access evidence digitally. Furthermore, the survey was useful in the current study context and efforts were made to increase survey construct and face validity by including stakeholders in survey development to ensure clarity of questions and comprehensiveness of the survey; however, the survey was not formally psychometrically validated. It is noted that psychometric validation of needs assessment surveys is uncommon within the needs assessment literature^54,55^; however, this does limit the use of the survey in contexts outside of that investigated in this study. Finally, this sample was primarily made up of Canadian respondents. Given the structure of the Canadian health care system, especially relative to other countries without universal health care, the concerns and needs expressed by those in the present sample may not entirely generalize to stakeholders that interact with a different style of health care system. Furthermore, access to resources via health care institutions or even via media (e.g., Internet, social media, etc.) may differ among countries, thus potentially limiting the generalizability of the current findings to international audiences in terms of preferred methods of accessing evidence. Future research should explore the barriers and needs of stakeholders in diverse social and geographical contexts to further our understanding of common and unique needs of stakeholders when it comes to access to resources for children’s pain management.

## Conclusion

Stakeholders face similar barriers to accessing evidence-based resources on children’s pain management but report diverse needs and desired approaches to access this information and facilitate its implementation. Identification of these common factors and differences is critical to inform how evidence-based resources can be tailored to present information to various stakeholder groups in the most effective method possible. Furthermore, stakeholders identify a clear need for guidance on how evidence can be implemented in their specific contexts. Engaging stakeholders in knowledge production, resource development, and dissemination is a clear desire to ensure the resources created best serve their needs and can be easily accessed.

## Supplementary Material

Supplemental Material

Supplemental Material

## Data Availability

The data sets used and/or analyzed during the current study are available from the corresponding author on reasonable request.
