# Supplementary material for: Bridging the gap: Identifying diverse stakeholder needs and barriers to accessing evidence and resources for children’s pain
Source: Can J Pain. 2022 May 17;6(1):48–64. doi: 10.1080/24740527.2022.2045192 (PMC9116405; doi:10.1080/24740527.2022.2045192)
Supplement: Supplemental Material [file UCJP_A_2045192_SM1304.docx]

| **General Category** | **Description** | |
| --- | --- | --- |
| **Differences in Types of Evidence-Based Resources Used by Stakeholder Type** | Participants listed a range of resources available to various stakeholders, including patients and families, as well as researchers and health professionals. | |
| **Subcategory** | **Description** | |
| Electronic resources intended for patient/family resources | E.g., apps, social media, videos, websites | |
| Literature intended for patients/families | E.g., pamphlets, infographics, posters, other written resources | |
| Clinician resources | E.g., clinical practice guidelines, CAPHC resources | |
| Clinical tools | E.g., Buzzy, pain assessment questionnaires, Holland Bloorview Chronic Pain Assessment Toolbox, Comfort Ability | |
| Knowledge mobilization initiatives | E.g., Naitre et Grandir, It Doesn’t Have to Hurt, TREKK Resources, Immunize Canada | |
| Training opportunities | E.g., knowledge sharing events, scientific cafes, training sessions | |
| **General Category** | **Description** | |
| **Barriers to Accessing/Implementing Evidence-Based Resources for Children’s Pain** | Participants described a range of barriers to accessing evidence-based resources, including knowledge of available resources, challenges gaining access to resources, and systemic barriers to implementation of information from resources. | |
| **Subcategory** | **Description** | **Example Quotation** |
| Challenges locating and accessing relevant information | Participants described challenges knowing where to look for evidence-based resources, especially for specific health issues and patient groups (e.g., palliative care), or in languages other than English. When participants knew where information could be located, they reported challenges gaining accessing to it due to barriers such as restricted access journals. | *[There is a] lack of access to journal articles, unless open.*  *There is information on the web that is not evidenced based but those without research skills may think it is and implement these practices due to easy of understanding.* |
| Lack of knowledge and support for accessing and implementing evidence-based resources | There is insufficient leadership initiative on the part of various stakeholders to identify and implement evidence-based resources for children’s pain. Participants report that barriers are exacerbated when institutional policies limit access and resources to implementing these practices and resources. Caregivers also cite insufficient knowledge on rights to pain management, creating barriers to accessing and implementing evidence-based practices to manage children’s pain within their own families. | *[There is a] lack of leadership and knowledge in others.*  *Hospital policies related to nurse initiated pathways and challenges with forms.* |
| **General Category** | **Description** | |
| **Needs to Ensure Use of Evidence-Based Resources** | In order to ensure implementation of evidence-based practices, practical needs were identified, such as resources and institutional support, as well as systemic supports, such as policies and cultural recognition of the importance of pain management. Partnerships were identified as a key mechanism to increase the visibility and availability of evidence-based resources, as well as to develop practical implementation plans as informed by multiple stakeholders. | |
| **Subcategory** | **Description** | **Example Quotation** |
| Supportive strategies for effective evidence-based practice implementation | Participants reported that support was required to effectively implement evidence-based practices, including practical resources (e.g., staff to deliver interventions, clinical practice guidelines, implementation evaluation guidance), tailored supports (e.g., language and/or population specific resources), and training opportunities for care providers (e.g., education for health professionals and trainees to learn about effective pain management strategies and how to implement them, funding for implementation). | *Hire and evaluate service leaders (admin and clin) that will apply evidence and evaluate their performance accordingly.*  *Assisting non-pediatric hospitals especially non 'big city' hospitals implement best practices without taxing their often limited resources (people and things).*  *Tools for all non-English speakers. Our second most commonly spoken language in [city] is Arabic. Huge need!* |
| Broader awareness of, and support for, pain management practices and implementation | Recognizing the value of pain management was seen as a crucial culture shift to increase implementation of evidence-based practices, necessary to be accompanied by institutional administrative policy changes to support implementation. Broader awareness was also identified as a need, with calls to promote knowledge of evidence-based practices for pain management in the broader public. | *Engaging parents in raising public awareness [of children’s pain management].*  *Changing the culture of the under treatment of pain.* |
| Partnerships to promote development of knowledge mobilization initiatives | Stakeholder engagement was identified as central to resource development to support knowledge mobilization efforts. Participants also described partnerships as necessary not only for creating resources, but to inform effective implementation strategies across specific contexts. | *Focus on multi-stakeholder knowledge translation initiatives that seek to identify primary barriers to implementing evidence-based solutions and co-design interventions to overcome those barriers.* |
| **General Category** | **Description** | |
| **Interest in knowledge mobilization activities related to children’s pain** | Respondents shared a range of interests regarding participation in activities related to the dissemination and implementation of evidence-based resources for children’s pain management. These included engaging in implementation, receiving resources and training, or sharing their perspectives to inform the development of initiatives and resources. | |
| **Subcategory** | **Description** | **Example Quotation** |
| Dissemination and implementation activities | Participants reported interest in engaging in dissemination and implementation activities by implementing practices within in clinics, sharing resources with relevant networks, providing consultation for implementation, and promoting awareness of evidence-based resources. Participants also reported interest in receiving resources (both individual resources and access to repositories) as well as educational and training opportunities. | *Happy to support collation of evidence into a central accessible source and distribute access/knowledge about this source internationally beyond Canada.*  *Interested in the implementation and promotion of tools; spreading the word through our networks.* |
| Generating knowledge and resources | Participants expressed interest in contributing to research and resource development, in terms of research participation (both as investigators and participants) and in terms of creation of resources to then be shared, including tools in different languages. | *Would be interested in identifying evidence-based knowledge but beyond that to develop materials to share what is relevant in an accessible way to other stakeholders (families healthcare providers administration).* |
| Sharing perspectives as a stakeholder | Other participants expressed a desire to be engaged in their capacity as a specific stakeholder, to share their unique perspective as it related to various research, development, dissemination, and implementation opportunities. | *My role is split between producing original research and leading knowledge mobilization activities at our hospital. I would like to bring the perspective of children with pain who have intellectual and developmental disabilities.* |
| **General Category** | **Description** | |
| **Other Comments on Future KMb Needs and Directions in Children’s Pain** | Participants described a need for pain not only to be managed more effectively at the clinical level, but identified several pathways to achieve better implementation of evidence-based practices. These include partnership and collaboration across stakeholders, as well as addressing the need for resources for individuals who have diverse needs or socio-cultural contexts. | |
| **Subcategory** | **Description** | **Example Quotation** |
| Need for pain to be recognized and treated seriously | Participants described the need for a culture shift toward recognizing the importance of managing children’s pain and having this be a priority in clinical care. SKIP was recognized as a key initiative toward shifting the culture toward making pain management a priority. | *This is a very important initiative. Pain is under recognized and under treated in children with physical disabilities. Easier access to up to date evidenced based assessment tools and management guidelines are needed to ensure our population is better managed.* |
| Need for partnership to advance pain management efforts | A clear desire for partnership was expressed with the goal of increasing collaboration between diverse stakeholders, including health and research experts, as well as patient partners. Interdisciplinary collaboration was also identified as a need in order to create effective and relevant KMb materials. | *Involve dentists please. They can learn from your knowledge base (e.g. how children respond differently to pain than adults). Since they are part of the opioid problem dentists learning about appropriate pain management is essential.*  *Present at Association for Child Life Professionals Conference or local Child Life conferences as we often are the go-to for non-pharmacological pain management.* |
| Need for more knowledge and resources | Overall, there was a strong indication from respondents that there is a greater need for research on how to implement evidence-based resources, as well as education for those who be engaged in implementation. Additionally, respondents indicated a clear need for resources intended for underrepresented groups, including children with special needs, marginalized groups, and availability of resources in a range of languages. | *I would love to see tools developed for refugee families and their children. And the doctors who care for them. Beyond seeing professionals at the [hospital] there is almost no culturally appropriate pediatric care for refugee children in the Maritimes.* |
