# Supplementary figures and images for: Bridging the gap: Identifying diverse stakeholder needs and barriers to accessing evidence and resources for children’s pain
Source: Can J Pain. 2022 May 17;6(1):48–64. doi: 10.1080/24740527.2022.2045192 (PMC9116405; doi:10.1080/24740527.2022.2045192)

**Online Needs Assessment Survey**


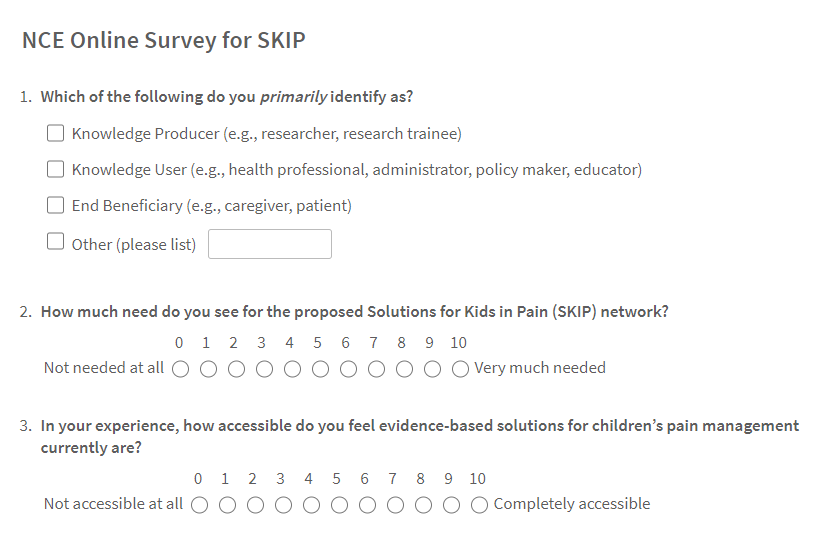


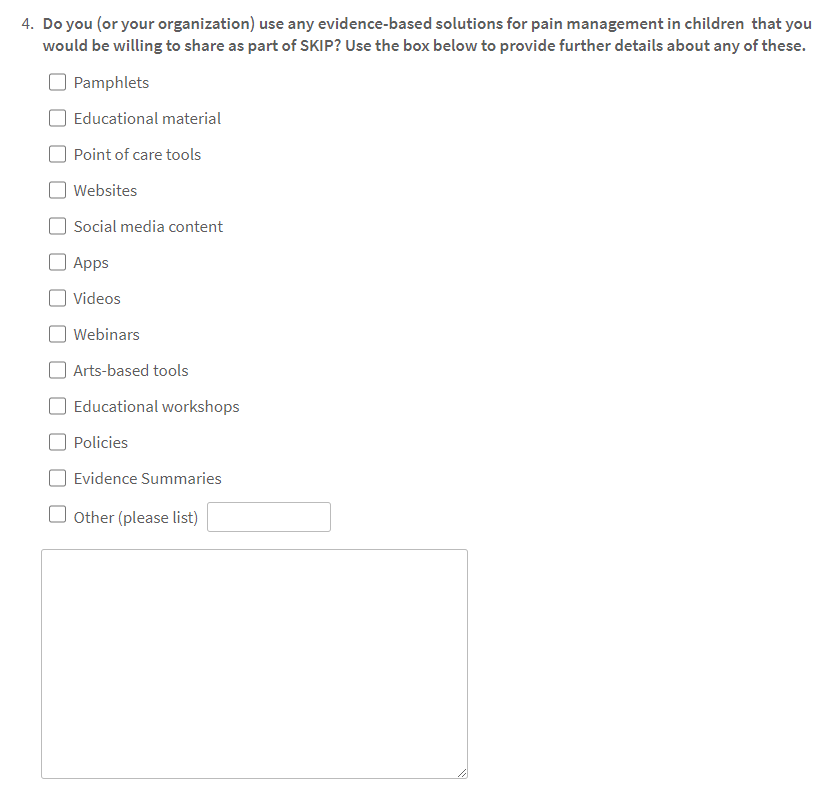


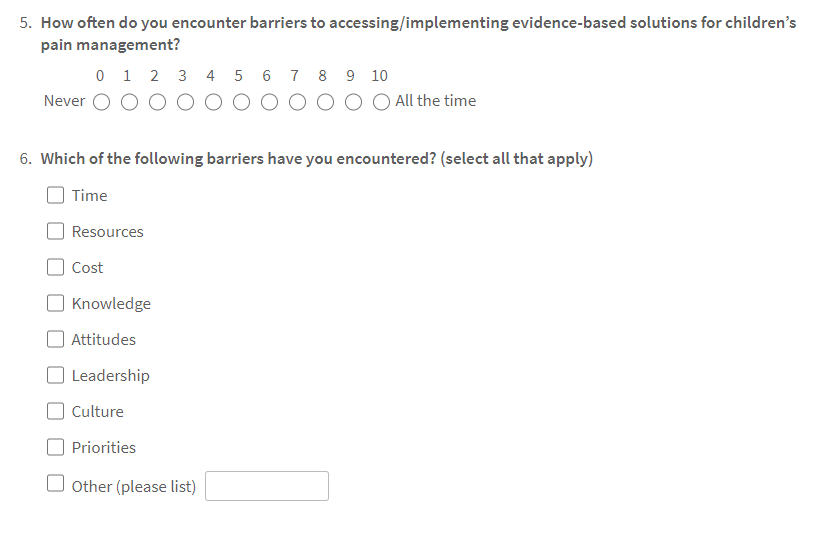


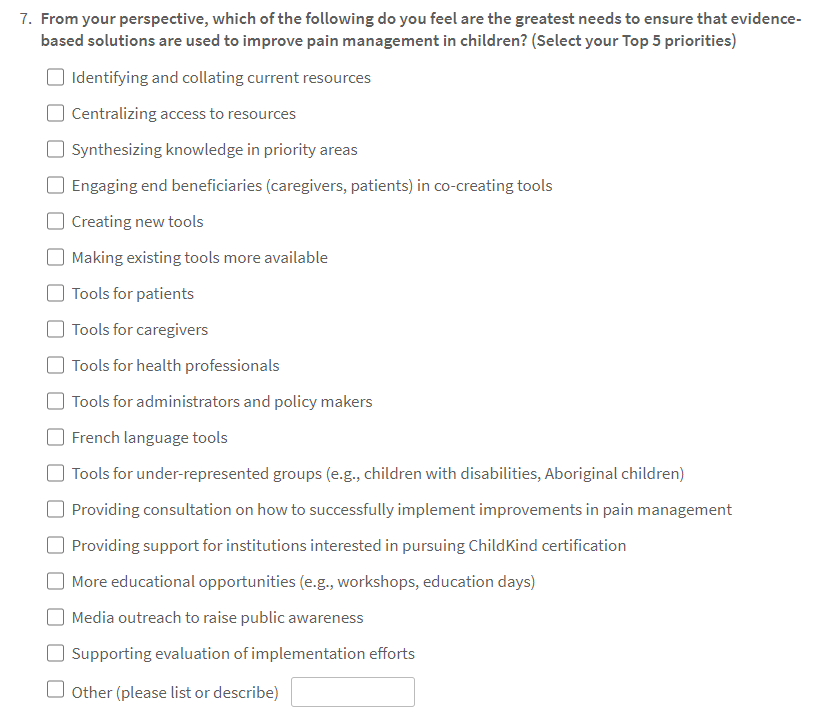


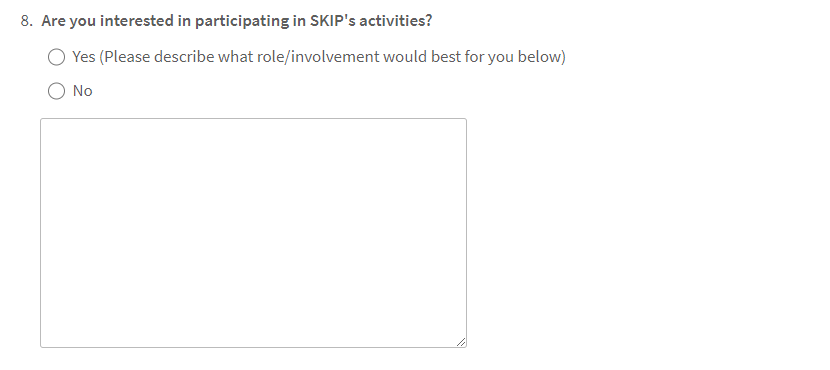


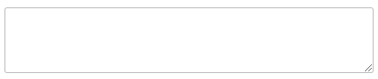


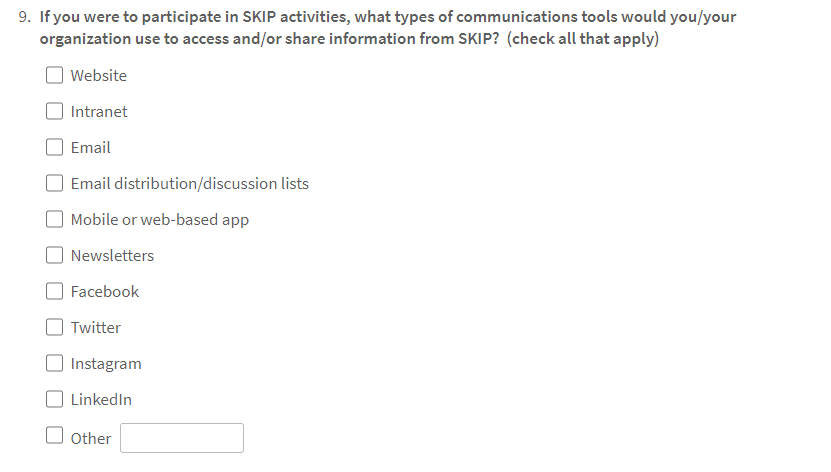

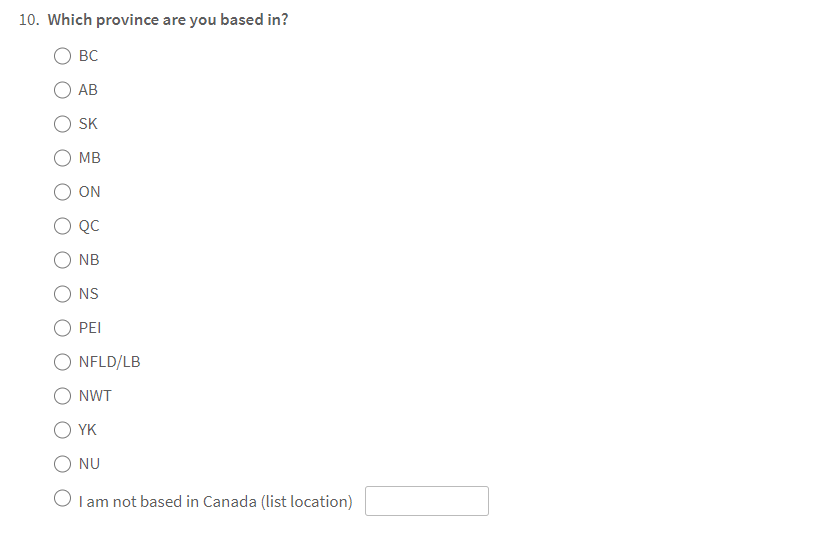


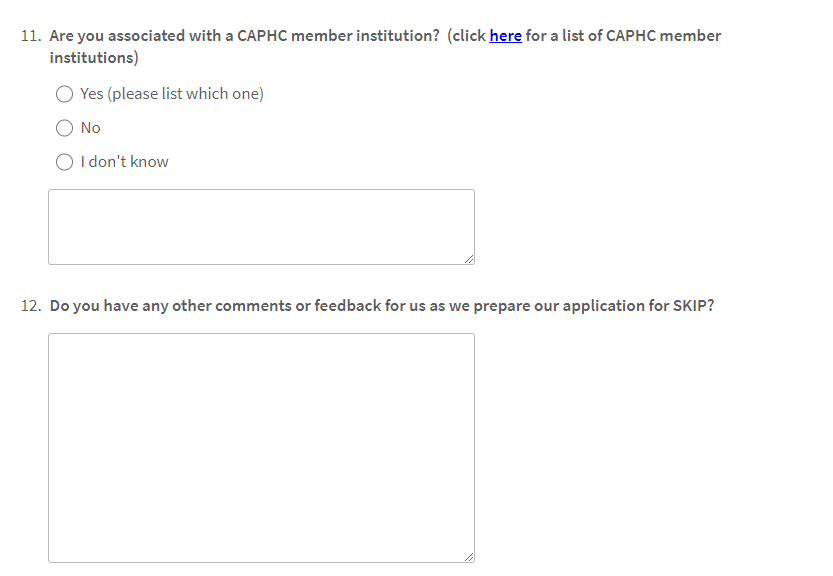

Supplement: Supplemental Material [file UCJP_A_2045192_SM1299.docx]
